# The Physiology Of the WEight Reduced State (POWERS) study: design and rationale for assessment of food intake, physical activity and other behavioral constructs

**DOI:** 10.1038/s41366-025-01991-3

**Published:** 2026-01-09

**Authors:** Laurel E. S. Mayer, Michael Lowe, Kelly C. Allison, Maxine Ashby-Thompson, Giada Benasi, Kyle S. Burger, Roger A. Fielding, Karin Foerde, Dympna Gallagher, John M. Jakicic, Matthew R. Hayes, Christopher E. Kline, Maren R. Laughlin, Susan B. Roberts, Marie-Pierre St-Onge, Kathryn J. Whyte, Susan Z. Yanovski, Deborah Young-Hyman, Wendy C. King

**Affiliations:** 1 Department of Psychiatry, Columbia University Irving Medical Center, New York, NY USA; 2 Department of Psychological and Brain Sciences, Drexel University, Philadelphia, PA USA; 3 Department of Psychiatry, Perelman School of Medicine, University of Pennsylvania, Philadelphia, PA USA; 4 Division of Endocrinology, New York Nutrition Obesity Research Center, Columbia University, New York, NY USA; 5 Department of Pediatrics, Molecular Genetics, College of Physicians and Surgeons, Columbia University, New York, NY USA; 6 Department of Medicine, Division of General Medicine, Columbia University Irving Medical Center, New York, NY USA; 7 Department of Psychology, Rowan University, Glassboro, NJ USA; 8 Department of Nutrition, Gillings School of Global Public Health and School of Medicine, University of North Carolina at Chapel Hill, Chapel Hill, NC USA; 9 Nutrition, Exercise Physiology, and Sarcopenia Laboratory, Jean Mayer USDA Human Nutrition Research Center on Aging at Tufts University, Boston, MA USA; 10 Department of Psychology, Brain and Cognition, University of Amsterdam, Amsterdam, The Netherlands; 11 Department of Internal Medicine, Division of Physical Activity and Weight Management, University of Kansas Medical Center, Kansas City, KS USA; 12 Department of Health and Human Development, School of Education, University of Pittsburgh, Pittsburgh, PA USA; 13 Division of Diabetes, Endocrinology and Metabolic Diseases, National Institute of Diabetes and Digestive and Kidney Diseases, National Institutes of Health, Bethesda, MD USA; 14 Dartmouth College Geisel School of Medicine, Hanover, NH USA; 15 Center of Excellence for Sleep & Circadian Research, Columbia University Irving Medical Center, New York, NY USA; 16 Division of Digestive Diseases and Nutrition, National Institute of Diabetes and Digestive and Kidney Diseases, National Institutes of Health, Bethesda, MD USA; 17 Office of Behavioral and Social Sciences, Office of the Director, National Institutes of Health, Bethesda, MD USA; 18 Department of Epidemiology, School of Public Health, University of Pittsburgh, Pittsburgh, PA USA; 19 Department of Quantitative Health Sciences, Cleveland Clinic, Cleveland, OH USA; 20 Columbia University, New York, NY USA; 21 AdventHealth Research Institute, Orlando, FL USA; 22 Tufts University, Boston, MA USA; 23 University of Pennsylvania, Philadelphia, PA USA; 24 University of Aberdeen, Aberdeen, UK; 25 Drexel University, Philadelphia, PA USA; 26 University of Pittsburgh, Pittsburgh, PA USA; 27 University of Florida, Gainesville, FL USA; 28 University of Rochester, Rochester, NY USA; 29 Office of Disease Prevention, National Institutes of Health, Bethesda, MD USA

**Keywords:** Physiology, Clinical trial design

## Abstract

The Physiology Of the WEight Reduced State (POWERS) study is a multi-center NIH-funded clinical trial designed to determine the physiological basis for variability in weight loss maintenance among adults with obesity following participation in a behavioral weight loss program. Two hundred and five healthy adults, aged 25–<60 years, with body mass index 30–<40 kg/m^2^ complete up to four serial assessments (before weight loss; after ≥7% weight loss; and four and 12 months later). This report, one in a five-part series on the POWERS study design, provides the rationale for and description of behavioral measures. Standardized laboratory meals are used to measure energy intake and eating-related behaviors. Behavioral and neurocognitive factors related to eating (e.g., food-choice decision making, taste preferences, reward, self-control) are assessed via computer-based tasks and self-report questionnaires. Functional and structural neuroimaging augment the behavioral assessments by identifying underlying neural circuitry. Psychological factors related to weight regulation (e.g., self-monitoring, stigma, self-efficacy) are assessed via self-report questionnaires. Free-living physical activity and sleep are measured via accelerometry, polysomnography and self-report questionnaires. We will evaluate how changes, integrated values and patterns in these predictors and components of energy intake and energy expenditure contribute to individual variability in weight change during the 12 months following weight loss. We anticipate that extensive phenotyping using sophisticated eating behavior paradigms and assessments of critical components of energy expenditure before and after weight loss will lead to improved predictions of successful weight loss maintenance. This, in turn, will inform more effective treatments for long-term sustained weight loss.

## Introduction

Weight regain after intentional weight loss is common and occurs when energy intake (EI) exceeds energy expenditure (EE) over time. The Physiology Of the WEight Reduced State (POWERS) study is a multi-center NIH-funded clinical trial designed to determine the physiological basis for variability in weight loss maintenance among adults with obesity following participation in a behavioral weight loss program. Through extensive phenotyping, biospecimen collection, and qualitative assessments, the POWERS study aims to identify pathways and predictors of the adaptations that occur after weight loss and their contributions to the heterogeneity of maintenance of reduced weight. An overview paper describes the rationale for and history of the POWERS study, as well as the study organization and overarching design [1]. In this report, we provide the rationale for and methods used to assess behavioral measures, specifically, measures of food intake and related eating- and weight-related cognitions, affect and behaviors; and free-living physical activity and sleep. Functional and structural neuroimaging augment the behavioral assessments by identifying underlying neural circuitry. Additional POWERS study design papers describe measures of energy balance [2], biomarkers [3] and environmental, social and psychosocial determinants of health [4].

## Methods

A complete description of the study design is provided in the POWERS study overview paper [1]. Briefly, 205 healthy adults, aged 25–<60 years, with class 1 and 2 obesity (body mass index [BMI] 30–<40 kg/m^2^) are being enrolled from two sites, Columbia University Irving Medical Center (CUIMC) and Drexel/Dartmouth/Tufts/Penn (DTP), with the goal to obtain a sample that is ≥40% male, ≥40% racial or ethnic minority and balanced across the eligible age range. Participants will be evaluated at up to four timepoints for a period of 16 days each. The baseline (BL) assessment is conducted after a period of documented weight stability prior to starting a behavioral weight loss intervention. Participants who lose at least 7% of their body weight during the intervention are next assessed after achieving weight stabilization for at least 2 weeks (time zero [T0]), and then followed observationally (i.e., without intervention) for 1 year, with assessments at four (T4) and 12 (T12) months.

A schedule of the procedures described in this paper is provided in Table 1. Except for the functional magnetic resonance imaging (fMRI) scans, procedures are identical at both sites. Because some procedures involve consuming standardized laboratory meals, following a severely restricted diet (e.g., vegan, very low carbohydrate and gluten-free) and having a known severe food allergy are exclusion criteria. Please see the overview paper [1] for a listing of all inclusion and exclusion criteria. This trial is registered at clinicaltrials.gov as NCT05748158.

**Table 1:** The schedule of behavioral measures in the POWERS study.

Variable(s) of interest	Procedure	Timepoints			
		BL	T0	T4	T12
**Energy intake and eating-related behaviors**					
Food intake, macronutrient distribution, diet energy density, diet variety	Multiple-item Test Meal		x	x	
Hedonic eating	Eating in the Absence of Hunger laboratory meal and snack		x	x	
Healthiness and tastiness preference and food choice	Food Choice Task	x	x	x	x
Neural circuits engagement during food choice	Food Choice Task during functional MRI (at CUIMC only)		x	x	
Learning and reward circuit engagement during palatable tastes and their anticipatory cues	Taste and Anticipatory Cue Gustatory Task during functional MRI (at DDPT only)		x	x	
Midbrain dopamine levels	Neuromelanin-sensitive MRI		x	x	
White matter tractography and connectivity	Diffusion-weight MRI		x	x	
**Psychological factors related to eating behavior and weight**					
Weight-related self-monitoring	Weight Control Strategies Scale - self-monitoring subscale items		x	x	x
Eating patterns	Action for HEAlth in Diabetes (Look AHEAD) Eating Patterns Questionnaire	x	x	x	x
Meal replacement use	Meal replacement frequency question		x	x	x
Binge and loss-of-control eating	Questionnaire on Eating and Weight Patterns-5	x	x	x	x
Dietary restraint, disinhibition and hunger	Three Factor Eating Questionnaire-51 item version	x	x	x	x
Food preferences	Geiselman Food Preference Questionnaire	x	x	x	x
Stress or emotional eating	Palatable Eating Motives Scale—Coping subscale	x	x	x	x
Food craving	Food Craving Questionnaire-Trait-Reduced	x	x	x	x
Hedonic hunger	Power of Food Scale	x	x	x	x
Weight stigma	Weight Bias Internalized Scale	x	x		
Weight loss goals	Goals and Relative Weight Questionnaire	x			
Confidence in weight loss maintenance	Confidence in Weight Loss Maintenance Questionnaire		x		
**Free-living physical activity**					
Objective steps, activity counts and time in sedentary behavior and activity by intensity	ActiGraph GT3X+ (wrist-worn actigraphy)	x	x	x	x
Self-report time in moderate and vigorous activity by domain	Global Physical Activity Questionnaire	x	x	x	x
**Sleep**					
Objective sleep duration, efficiency, latency, timing, wake after sleep onset, frequency of awakenings, and ambient light	ActiGraph GT3X+ (wrist-worn actigraphy)	x	x	x	x
Objective sleep quality and disturbances (e.g., apneas and hypopneas)	zMachine Synergy System (polysomnography)	x	x	x	x
Self-report sleep quality	Pittsburgh Sleep Quality Index	x	x	x	x
Self-report chronotype	Morningness-Eveningness Questionnaire	x	x	x	x

*BL* baseline, *CUIMC* Columbia University Irving Medical Center, *DTP* Drexel/Dartmouth/Tufts/Penn, *MRI* magnetic resonance imaging, *T0* time zero, *T4* time 4 months, *T12* time 12 months.

### Ethics approval and consent to participate

The protocol and informed consent forms were approved by the single Institutional Review Board at the University of Pittsburgh (CR21120046-021). All participants will undergo informed consent prior to enrolling in the study described in this manuscript, and all methods will be performed in accordance with the Declaration of Helsinki.

### Energy intake and eating-related behaviors

#### Direct observation of food intake

Measures of average total daily EI by doubly labeled water and self-reported dietary intake by serial 24-h recalls are described in the POWERS energy balance paper [2]. While self-reported dietary recalls measure food intake in a naturalistic setting and allow for nuanced evaluations of dietary intake, they are prone to individual recall and social desirability bias; furthermore, individuals with obesity underreport EI to a greater extent than individuals with healthy weight [5]. To address these limitations, two assessments of food intake under controlled laboratory conditions are conducted at T0 and T4. Foods and beverages used in these tests are prepared and processed similarly by the staff of the Bionutrition Research Core of the Clinical and Translational Science Award program at each clinical site.

##### Multiple-item test meal

This study allows for the direct evaluation of food intake, including macronutrient distribution and other relevant nutritional constructs (e.g. diet energy density, diet variety), and eating behaviors during a standardized test meal [6]. Although limited to a single meal, it is sensitive to change in food intake over time in individuals with eating disorders [6].

In preparation for the test meal (dinner), participants are provided with a standardized 2092 kj (500 kcal) lunch consisting of a turkey sandwich with mustard, Nutrigrain bar and water (20% protein, 18% fat, 62% carbohydrates), which they are asked to fully consume within 20 min. Following lunch, participants abstain from food or drinks, except unflavored water, until the test meal, five hours later. Between lunch and the test meal, participants engage in the neuroimaging procedures (described below).

The test meal includes a range of entrees, sides, desserts, condiments and caloric and non-caloric beverages (Fig. S1) totaling approximately 62,760 kj (15,000 kcal; 19% protein, 44% fat, 38% carbohydrates). Participants are instructed, “This is your dinner for today. Eat as much or as little as you like. You have up to 1 h to complete the meal.” For safety and to confirm that foods are consumed and not otherwise disposed of, participants are informed that the test meal is observed via non-recorded video by research staff. Participants rate their hunger, fullness, and desire to eat on 100-mm visual analog scales three times: (1) prior to seeing the available foods (outside the eating lab), (2) after seeing the available foods but before eating, and (3) right after the meal. Additionally, right after the meal, participants are asked to estimate the number of calories consumed and to rate their feeling of a loss-of-control during the meal. Each food item is weighed prior and subsequent to determine consumption.

##### Eating in the absence of hunger

The construct of eating in the absence of hunger is evaluated on a separate day than the Multiple-item Test Meal via a standardized test which measures the amount of highly palatable snack foods consumed following a standardized lunch. The additional calories consumed in the presence of recent energy intake may be a proxy of hedonic intake as well as a behavioral indicator of loss-of-control vulnerability, which may be associated with weight regain, independent of total caloric intake [7].

After fasting from the night before until lunch time, participants are asked to consume a standardized 2092 kj (500 kcal) portion of macaroni and cheese (18% protein, 40% fat, 42% carbohydrates) within 15 min. Then three bowls with peanut butter cups, tortilla chips, and salted almonds, respectively (totaling 11,987 kj/2865 kcal; 10% protein, 66% fat, 31% carbohydrates), water, and taste-rating sheets are brought into the room (Fig. S2). Participants are given the following instructions: “We want to assess a variety of your food preferences as part of the study. Now that you have had a meal, we are going to test your preferences for several snack foods. Help yourself to whatever you like, as long as you at least taste each of the three types of snacks.” Participants have 10 min to sample and rate the taste of the snack foods. Participants rate their hunger, fullness, and desire to eat on 100-mm visual analog scales five times: (1) outside the eating lab, (2) after the meal is delivered and meal instructions are given but before food is eaten, (3) right after the 15-min meal consumption period, (4) right after snacks are brought into the lab, and (5) when the snack-tasting phase is over. Additionally, participants are asked to rate their feeling of a loss-of-control during the snack period (i.e., test). Each snack bowl is weighed prior and subsequent to the test to determine caloric consumption.

##### Food valuation, reward and decisions: exploring neural mechanisms

Although the behaviors that support weight loss maintenance may be guided initially by the conscious exertion of self-control [8], over time, these behaviors may become less effortful and/or more automatic [9]. Additionally, distinct neural reward responses to palatable tastes and their anticipatory cues in individuals with obesity [10] and those at risk for obesity [11] have been associated with future weight gain. Moreover, these response patterns are sensitive to dietary intervention [12, 13]. As behaviors can be mediated by different neural systems (e.g., goal-directed, reward or habitual) [14, 15], it is important to understand how these systems are associated with dieting behavior and weight loss maintenance.

##### Food Choice Task

This computer-administered task is completed behaviorally at all timepoints to provide information about how individuals assess the value of food (e.g. healthiness and tastiness) and make choices of what to eat (including the engagement of self-control). At the T0 and T4 assessment points, the Multiple-item Test Meal occurs, thereby linking food choice in the task with actual food intake [16]. Also, at the T0 and T4 timepoints, at the CUIMC site, the task is conducted a second time during fMRI scanning. This repeated assessment over time may allow us to identify if and when a shift in neural systems engaged in food choice occurs, potentially related to a change from goal-directed (engaging self-control) towards automatic/habitual control of behavior, thus elucidating the mechanisms associated with successful weight loss maintenance.

Participants complete the Food Choice Task approximately 2 h after eating their usual lunch at home. The task, which has three blocks, displays images of food. During the tastiness and healthiness blocks, participants are asked to rate 76 food images for tastiness or healthiness, respectively, on a 5-pt Likert scale (“good” to “bad” or “healthy” to “unhealthy”, with a “neutral” middle option). The order of tastiness and healthiness blocks are counterbalanced and the order of 76 food images within each block are pseudo-randomized across participants. On completion of these blocks, an image of food that was rated as “neutral” for both tastiness and healthiness by the participant is automatically selected as the reference for the choice block. During the choice block, each of 75 other food images is displayed one at a time and the participant is instructed to choose whether they would hypothetically prefer to eat a snack-sized portion of the displayed food or the reference food following the task.

##### Taste and anticipatory cue gustatory task

At DTP, a well-established taste and anticipatory cue gustatory fMRI paradigm [17] is used at T0 and T4 to examine brain response to high-sugar/high-fat taste receipt, cue-elicited anticipation of taste and to evaluate heterogeneities in brain response over repeated exposures, i.e., appetitive Pavlovian conditioning, which may predict heterogeneities in weight regain. Participants receive two fluids, a palatable chocolate milkshake and a tasteless, odorless solution containing the main ionic components of saliva, through individual beverage tubes via a customized manifold, which is anchored to the scanner bed. First, participants are presented with cues that predict the taste receipt of the milkshake to assess brain response to cue-elicited anticipation of food reward receipt. Following previous protocols [18], the anticipatory cues alerting participants to the upcoming two tastes are paired with two, novel fractals.

##### Neuromelanin-sensitive MRI

As dopamine is a key neurotransmitter involved in both hedonic motivation and decision-making neural systems, we use neuromelanin-MRI measures to assess the concentration of neuromelanin, as a potential biomarker for weight loss maintenance. Neuromelanin is a pigment generated by the conversion of cytosolic dopamine to an iron-containing paramagnetic complex which accumulates in the midbrain (substantia nigra, ventral tegmental area). Crucially, neuromelanin-MRI signal has been validated in vivo as a biomarker for dopamine function using positron emission tomography [19]. The MRI scans of neuromelanin take advantage of cutting-edge technology to explore dopamine function after acute weight loss and its relation to 12 months weight loss maintenance.

##### Diffusion-weighted MRI

Diffusion imaging allows closer evaluation of the integrity of white matter tracts including fiber density and fiber bundle morphology. This will allow investigation of connectivity between various relevant brain regions probed by the food choice and cue/taste/reward tasks, as well as the increasingly relevant cerebellum [20].

### Psychological factors related to eating behavior and weight

Eating-related behaviors, influencers of food intake, weight stigma and weight loss expectations may affect attempted weight loss and weight loss maintenance. We measure these domains via self-report, when possible, with existing valid and reliable questionnaires, some of which are Accumulating Data to Optimally Predict Obesity Treatment (ADOPT) Core Measures [21, 22]. While self-report is methodologically problematic for a variety of well-documented reasons (e.g., memory distortions, self-serving biases), these measures likely capture variance in EI that may predict variance in weight regain beyond that accounted for by physiological measures [23].

#### Eating-related behaviors

##### Weight control strategies

Use of self-monitoring techniques has been repeatedly described as a useful weight control strategy [24]. Thus, at T0, T4 and T12, participants complete the 7-item self-monitoring subscale of the Weight Control Strategies Scale, which focuses on diet monitoring, exercise tracking, and self-weighing [25]. The subscale has good reliability (Cronbach’s alpha = 0.89) and is validated in a weight loss-seeking population; scores positively correlate with post-treatment weight loss [25].

##### The Action for HEAlth in Diabetes (Look AHEAD) Eating Patterns Questionnaire

The first five items of this questionnaire are administered to assess eating patterns and practices associated with weight [26, 27]. Specifically, the items measure frequency of eating breakfast, lunch, and dinner, total meals/snacks per day and frequency of eating at fast-food or non-fast-food restaurants by meal.

##### Meal replacement use

During the weight loss intervention (described in [1]), participants are provided with beverage and bar meal replacements to reduce their caloric intake, and taught that meal replacements may be a helpful weight loss maintenance tool [28]. Thus, POWERS study investigators created a single question to assess the frequency of meal replacement use framed for each timepoint (e.g., “since the start of the weight loss intervention,” at T0, “in the past 4 months,” at T4 and “in the past 8 months,” at T12).

##### Questionnaire on Eating and Weight Patterns-5

This 26-item self-administered screening instrument for binge-eating disorder and bulimia nervosa [29], was updated from the Questionnaire on Eating and Weight Patterns-Revised [30], an ADOPT Core Measures [21], using criteria from the *Diagnostic and Statistical Manual of Mental Disorders, Fifth Edition* [31]. Decision rules characterize positive responses for binge eating frequency, duration, distress, and presence or absence of compensatory behaviors to identify and differentiate symptoms of binge-eating disorder or bulimia nervosa, although diagnosis requires clinical interview. It also captures loss-of-control eating episodes without an objectively large amount of food (i.e., subjective binge episodes). In POWERS, questions assessing demographics (assessed elsewhere) and binge content were removed, leaving the 9 core items required for scoring, which can be used to track frequency of binge/loss-of-control eating episodes over time. These core items have predictive value for identifying eating behaviors that may attenuate weight loss during behavioral treatment [32].

##### Three-Factor Eating Questionnaire

This 51-item questionnaire (also called the Eating Inventory) was designed to assess three cognitive and behavioral domains of eating: cognitive restraint, disinhibition and hunger; each domain has sub-categories for clearer distinction [33]. The cognitive and behavioral domains assessed by the Three-Factor Eating Questionnaire have been associated with successful weight maintenance [34].

#### Influencers of food intake

##### Food Preference Questionnaire

This 72-item scale assesses participants’ foods preferences of varying fat content [35]. The scoring system calculates a fat preference score (FPS) for high- versus low-fat foods and mean hedonic ratings for each macronutrient combination. The FPS was positively correlated with energy and fat intake among men with wide ranging BMIs [35] and among women with and without anorexia nervosa [36]. In POWERS, we will compare the FPS to observed fat intake (in the Multiple-item Test Meal) to determine if differences between self-reported food preferences and actual food intake meaningfully relate to weight loss maintenance.

##### Palatable Eating Motives Scale

This 20-item scale was designed to measure motivation for the consumption of “tasty” foods and drinks [37]. It consists of four subscales measuring potential motivations for eating palatable foods: Social, Enhancement, Conformity, and Coping. The POWERS study includes only the four-item Coping subscale, i.e., eating tasty food to deal with problems and negative emotions, because it accounts for unique variance in BMI [37].

##### Food Cravings Questionnaire-Trait-Reduced

This ADOPT Core Measure [21] is a subset of 15 items derived from an earlier and longer version of the Food Cravings Questionnaire [38], which focuses on trait-related craving items to identify enduring tendencies towards food cravings. It has high internal consistency and has been negatively associated with dieting success, indicating it may represent a risk factor for over-consumption [39].

##### Power of Food Scale

The Power of Food Scale [40] is a 15-item self-report measure assessing hedonic hunger and appetitive drive to consume palatable food when not food deprived. It contains three empirically-derived factors reflecting motivation toward the environmental availability of palatable food that is not physically present (Food Available factor), the presence of palatable food that has not been tasted (Food Present factor) and the initial tasting of palatable food (Food Tasted factor). It has shown high internal consistency and adequate test-retest reliability in college students [40] and its factor structure was replicated in a treatment-seeking sample with obesity [41]. It has a variety of food-related psychological, behavioral, nutritional and biological correlates [42].

#### Weight stigma and weight loss expectations

##### Weight Bias Internalized Scale

At BL and T0, this 11-item scale is used to assess the extent to which individuals with overweight or obesity internalize negative weight stereotypes [43]. The scale has shown high internal consistency (Cronbach’s *α* = 0.90) and is associated with mood and eating disturbance [43], and worse weight-related outcomes in adults trying to lose weight [44].

##### Goals and Relative Weights Questionnaire

At BL, this four-item questionnaire is used to assess weight expectations in four dimensions: Dream, Happy, Acceptable, and Disappointed weight [45]. It can help identify the gap between participants’ weight loss expectations and professional recommendations and may be predictive of weight regain. Previous studies show realistic goals lead to better weight management outcomes [46].

##### Confidence in Weight Loss Maintenance

An individual’s confidence in their capacity to adhere to essential eating and physical activity behaviors during the active phase of a behavioral weight loss intervention is predictive of better dietary intake, physical activity and weight outcomes after a year [47]. Accordingly, the POWERS study investigators created a two-item questionnaire, Confidence in Weight Loss Maintenance, to gauge participants’ confidence at T0 in their ability to maintain their weight loss. First, they are asked, “A year from now, do you think your weight will be “lower (at least 3 lbs.),” “the same (within 3 lbs.),” or “higher (at least 3 lbs.)?” [3 lb = 1.36 kg]. Then they rate their confidence in maintaining the majority of their weight loss in the next year, as “not at all,” “slightly,” “somewhat,” “fairly,” or “very.”

### Free-living physical activity

Based on the extensive literature relating physical activity to weight status and weight loss [48], as well as weight loss maintenance and weight regain [49], we will examine whether various aspects of physical activity (type, intensity, frequency and duration) are related to weight loss maintenance. We will also examine whether physical activity is associated with other lifestyle behaviors for weight maintenance (e.g., dietary intake) to identify behavior clusters that may be associated with improved outcomes. Because objective and self-report measures of physical activity both have advantages [50], the POWERS study utilizes both.

#### Objective assessment of physical activity

##### ActiGraph GT3X+

The ActiGraph GT3X+ (ActiGraph Corp, Pensacola, Florida) activity monitor is used to measure physical activity and sleep objectively for periods of 14 days. The wrist-worn GT3X + , which includes a micro-electro-mechanical system based accelerometer, was selected because it is a valid measure of both physical activity and sleep, and was used in the National Health and Nutrition Examination Survey, [51] allowing us to compare POWERS data with a nationally representative sample.

The monitor is worn on the non-dominant wrist. Data are processed and scored using the ActiLife Data Analysis Software (version 6.13.1, ActiGraph, Pensacola, Florida). We are using the Freedson (1998) cut-points to estimate time in sedentary, light, moderate and vigorous intensity activity [52]. Accelerometry variables (listed in Table 2) are determined at the day level. Because recommended accelerometer decision rules may change over the course of the POWERS study, the definition of a valid day and minimum day requirement will be determined based on recommendations in the literature just prior to centralized data processing.

**Table 2:** Actigraphy and polysomnography variables collected in the POWERS study.

	Unit
**ActiGraph GT3X+ physical activity variables**	
Steps	Steps
Minutes of sedentary time in bouts ≥1 min	Minutes
Wear-time in sedentary behavior	Percentage
Sedentary time in bouts ≥1 min	Minutes
Light physical activity in bouts ≥1 min	Minutes
Moderate physical activity in bouts ≥1 min	Minutes
Vigorous physical activity in bouts ≥1 min	Minutes
Moderate-to-vigorous physical activity in bout ≥1 min	Minutes
Moderate-to-vigorous physical activity in bouts ≥10 min	Minutes
Activity counts in ≥10 min bouts	Activity counts
Vector magnitude counts	Vector magnitude counts
**ActiGraph GT3X+ sleep variables**	
Bedtime	Clock time (h:min)
Sleep onset time	Clock time (h:min)
Wake time	Clock time (h:min)
Time in bed	Minutes
Total sleep time	Minutes
Sleep efficiency	Percentage
Wake after sleep onset	Minutes
Awakenings	Number
Movement index	Number
Sleep fragmentation index	Number
Wear time	Minutes
**zMachine sleep variables**	
Time in bed	Minutes
Total sleep time	Minutes
Sleep efficiency	Percentage
Apnea-hypopnea index	Ratio (per hour of sleep)
Respiratory disturbance index	Ratio (per hour of sleep)
Oxygen saturation <89%	Cumulative minutes
Central apneas	Number
Obstructive apneas	Number
Apneas + hypopneas	Number
Mean oxygen saturation during sleep	Percentage
Mean heart rate	Beats per minute
Latency to persistent sleep	Minutes
Total sleep time in light sleep	Percentage
Total sleep time in deep sleep	Percentage
Total sleep time in rapid eye movement (REM) sleep	Percentage
Total sleep time in non-REM sleep	Percentage
Wake after sleep onset	Minutes

#### Self-reported physical activity

##### Global Physical Activity Questionnaire (GPAQ)

The Global Physical Activity Questionnaire, an ADOPT Core Measure [22], was developed by the World Health Organization for physical activity surveillance [53]. While it has low-to-moderate validity when evaluated against accelerometry, fitness, and anthropometric measures [54], unlike doubly labeled water or accelerometry, it provides valuable information about how physical activity and sedentary behavior are accumulated (i.e., via recreation, transportation and occupation), which may contribute to EE. Additionally, it is easy to use and has low participant burden. Modeled after previous studies, e.g., [55], we added 4 items to the original 16 items, to assess household physical activity.

### Sleep

Shorter sleep duration and disordered breathing are related to greater EI [56]. Additionally, sleep architecture (i.e., structure of normal sleep cycles and stages) is associated with markers of energy balance [57]. Studies further suggest the relationship between weight loss and sleep health may be bi-directional [58, 59]. The POWERS study hypothesizes that multiple dimensions of sleep will be related to both EE and EI following the behavioral weight loss phase of the POWERS study. Because previous studies have reported associations between sleep, measured both by self-report and objective measures, and regulation of body weight [60], the POWERS study utilizes both assessment modalities.

#### Objective assessment of sleep

##### ActiGraph GT3X+

The ActiGraph GT3X+ provides information on sleep duration, efficiency, latency (i.e., time to fall asleep), timing (bedtime and wake time), restfulness, wake after sleep onset, and frequency of awakenings. Throughout the period the GT3X+ is worn, participants use a sleep diary, modeled after the Consensus Sleep Diary [61], to record bedtime, wake time, time to sleep onset and number and duration of nighttime awakenings, as well as the number and duration of daytime naps.

As with GT3X+ physical activity data, sleep data are processed and scored using ActiLife software. During processing, files are manually edited using standardized procedures to identify in-bed intervals from the sleep diary. Sleep scoring utilizes the Cole-Kripke algorithm [62]. Sleep variables (listed in Table 2) are determined at the day level. Minimum wear time requirements will be determined based on the literature at the time of centralized data processing.

##### zMachine Synergy System

We use a 9-channel portable polysomnography and electroencephalogram device, the zMachine Synergy System (General Sleep Corporation, Cleveland, OH) [63], to record data relevant to sleep-disordered breathing and sleep architecture over a single night. The zMachine Synergy System includes a pressure transducer (respiratory airflow and snoring), thoracoabdominal respiratory inductance plethysmography belt (respiratory effort), pulse oximeter (oxygen saturation and HR), and 3-axis accelerometer (body position). Participants are instructed on electrode placement for in-home measurements during one night of their choice, representing a typical work or school night (not a free or weekend night). The zMachine software automatically scores sleep stages every 30 s (auto-score function). Because the device includes an accelerometer, information on body position and respiratory events by body position is available.

#### Self-reported sleep

##### Pittsburgh Sleep Quality Index

This 19-item questionnaire, which assesses sleep quality and disturbances over the past month [64], is the most widely used sleep questionnaire internationally, [65] and has been used in studies evaluating relationships between sleep and weight change [66]. The items are used to create a global score and 7 domain scores (sleep quality, latency, duration, efficiency, and disturbances, use of sleep medications and daytime dysfunction) [64].

##### Morningness-Eveningness Questionnaire

This 19-item questionnaire [67] assesses an individual’s chronotype, i.e., the time during the day and night at which one feels their best to perform various activities, which has been shown to be related to EI [68] and EE [69]. The total score is used to categorize chronotype as definite evening, moderate evening, intermediate, moderate morning or definite morning.

## Discussion

This suite of sophisticated eating behavior paradigms and critical components of energy expenditure that collectively contribute to the variability in weight change during the 12 months following a behavioral weight loss intervention, especially when paired with structural and functional neuroimaging, have the potential to identify behavioral and neural signatures associated with successful weight loss maintenance. The harmonization of findings acquired through the POWERS study provides an unparalleled opportunity to integrate behavior and brain circuits in the service of understanding mechanisms that may promote long-term weight loss success. The ultimate goal is to use these insights in future clinical settings and to develop new treatments to improve maintenance of lost weight.

## Supplementary information

Supplemental Material: Figure S1 and Figure S2
