# Supplementary material for: The Physiology Of the WEight Reduced State (POWERS) study: design and rationale for assessment of food intake, physical activity and other behavioral constructs
Source: Int J Obes (Lond). 2026 Jan 9;50(9):1883–90. doi: 10.1038/s41366-025-01991-3 (PMC13340862; doi:10.1038/s41366-025-01991-3)

Figure 1. The layout of foods, including a range of entrees, side dishes, desserts, condiments and beverages, and the place setting for the Multiple-item Test Meal.

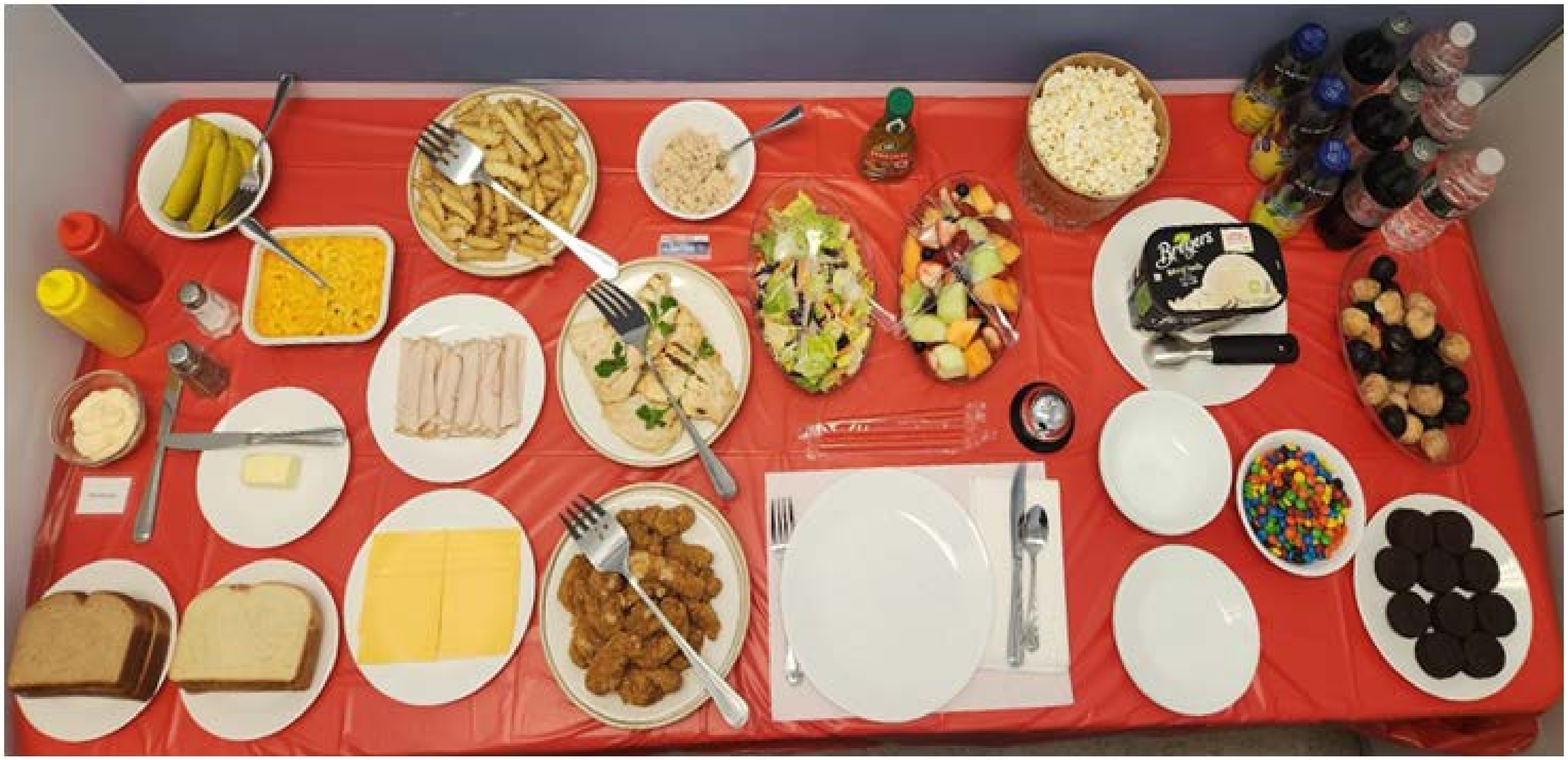

Figure 2. The layout of snacks (peanut butter cups, tortilla chips and salted almonds), water and taste-rating sheets given to participants following a standardized 2092 kj (500 kcal) lunch meal in the "Eating in the Absence of Hunger laboratory meal and snack."

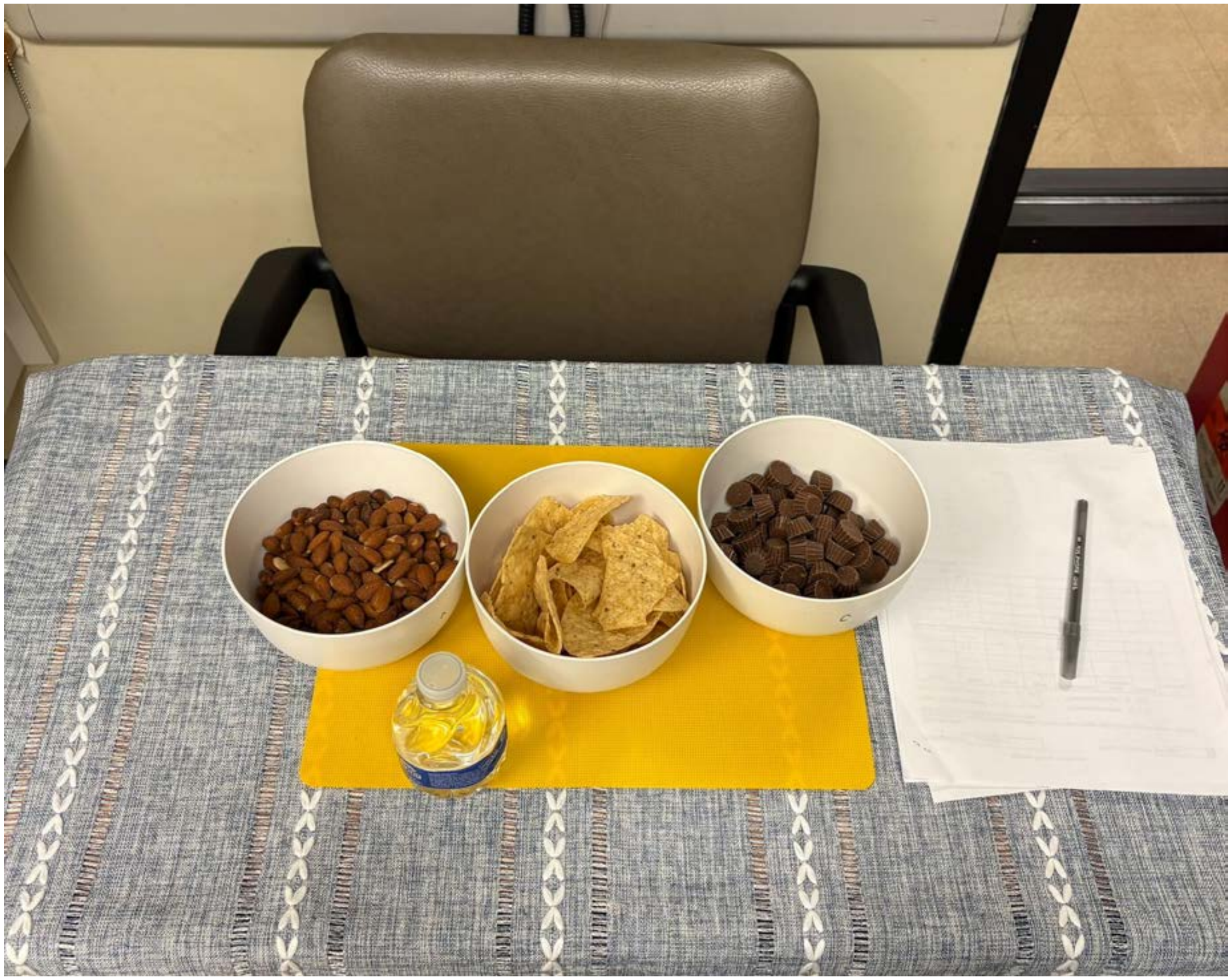

Supplement: Supplementary file 1 [file 41366_2025_1991_MOESM1_ESM.pdf]
