# Supplementary material for: The Physiology Of the WEight Reduced State (POWERS) study: design and rationale for assessment of food intake, physical activity and other behavioral constructs
Source: Int J Obes (Lond). Author manuscript; Available in PMC 2026 Jul 7. (PMC13340862; doi:10.1038/s41366-025-01991-3)

Figure 1. The layout of foods, including a range of entrees, side dishes, desserts, condiments and beverages, and the place setting for the Multiple-item Test Meal.

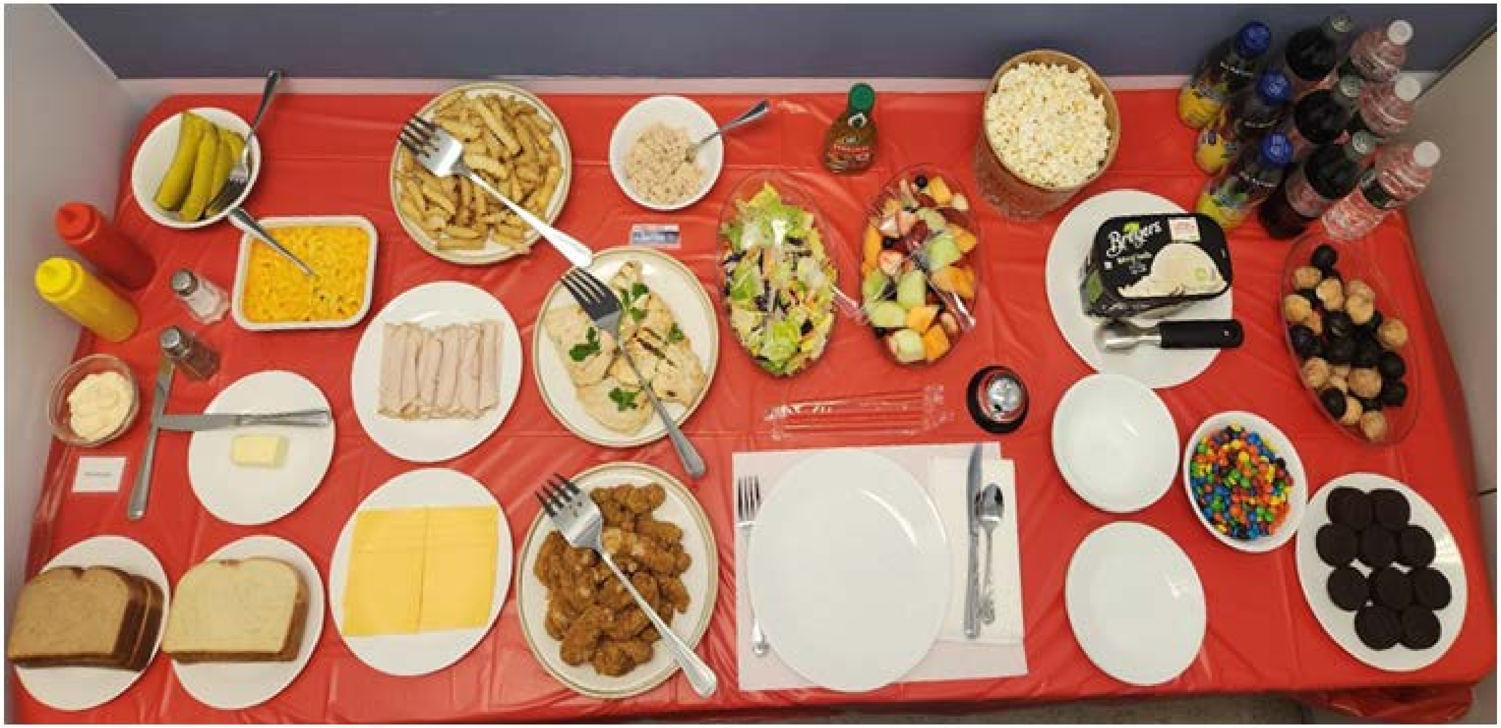

Figure 2. The layout of snacks (peanut butter cups, tortilla chips and salted almonds), water and taste-rating sheets given to participants following a standardized 2092 kj (500 kcal) lunch meal in the “Eating in the Absence of Hunger laboratory meal and snack.”

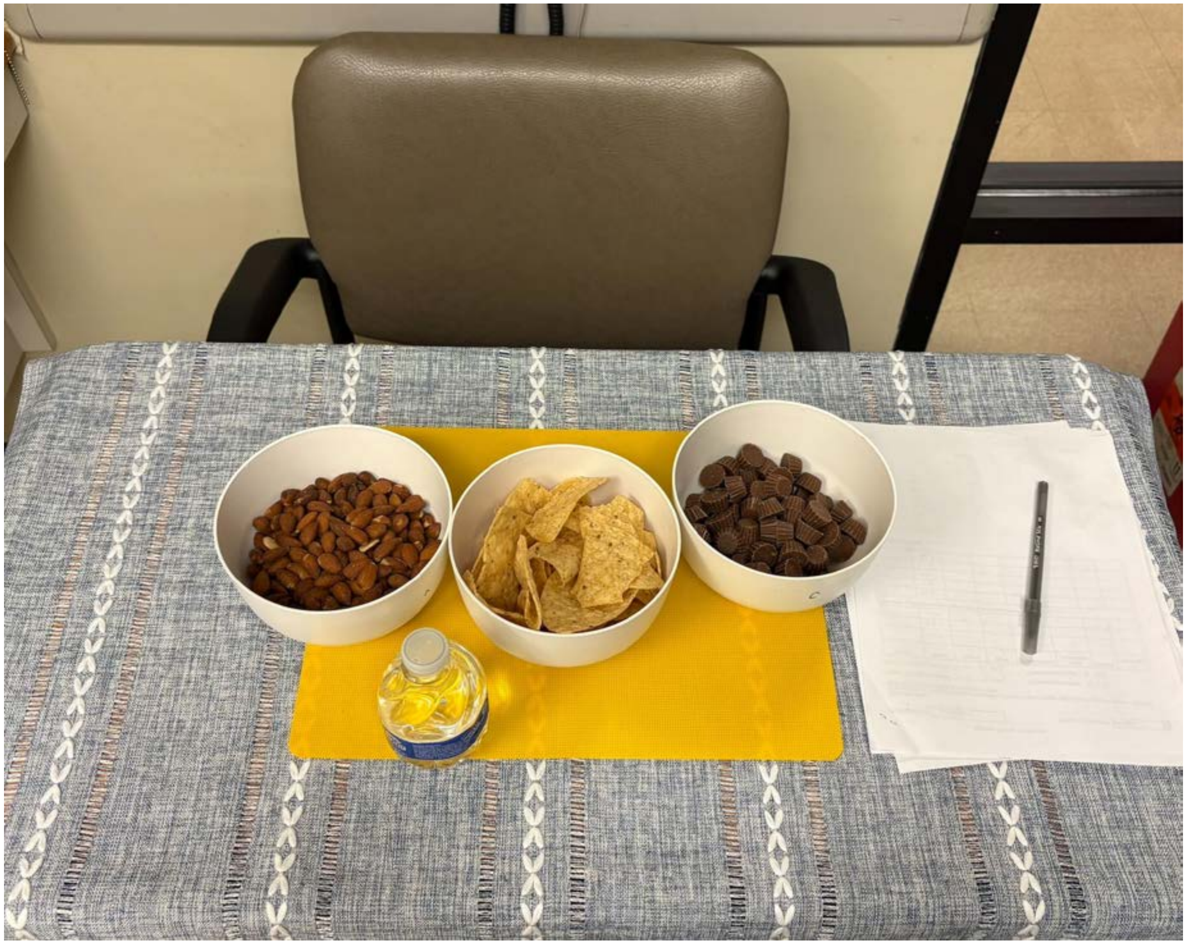

Supplement: Supplemental Figures 1 and 2 [file NIHMS2166521-supplement-Supplemental_Figures_1_and_2.pdf]
